# Genoviz Software Development Kit: Java tool kit for building genomics visualization applications

**DOI:** 10.1186/1471-2105-10-266

**Published:** 2009-08-25

**Authors:** Gregg A Helt, John W Nicol, Ed Erwin, Eric Blossom, Steven G Blanchard, Stephen A Chervitz, Cyrus Harmon, Ann E Loraine

**Affiliations:** 1 Genomancer Consulting, 9400 Mill Creek Road, Healdsburg, CA 95448, USA; 2 Dept of Bioinformatics and Genomics, University of North Carolina at Charlotte, 600 Laureate Way, North Carolina Research Campus, Kannapolis, NC 28082, USA; 3 Affymetrix, Inc 3420 Central Expressway, Santa Clara, CA 95051, USA; 4 Blossom Associates West, 2737 Russell St Berkeley, CA 94705, USA; 5 Olema Pharmaceuticals, Inc 665 3rd St, Suite 250, San Francisco, CA 94107, USA

## Abstract

**Background:**

Visualization software can expose previously undiscovered patterns in genomic data and advance biological science.

**Results:**

The Genoviz Software Development Kit (SDK) is an open source, Java-based framework designed for rapid assembly of visualization software applications for genomics. The Genoviz SDK framework provides a mechanism for incorporating adaptive, dynamic zooming into applications, a desirable feature of genome viewers. Visualization capabilities of the Genoviz SDK include automated layout of features along genetic or genomic axes; support for user interactions with graphical elements (Glyphs) in a map; a variety of Glyph sub-classes that promote experimentation with new ways of representing data in graphical formats; and support for adaptive, semantic zooming, whereby objects change their appearance depending on zoom level and zooming rate adapts to the current scale. Freely available demonstration and production quality applications, including the Integrated Genome Browser, illustrate Genoviz SDK capabilities.

**Conclusion:**

Separation between graphics components and genomic data models makes it easy for developers to add visualization capability to pre-existing applications or build new applications using third-party data models. Source code, documentation, sample applications, and tutorials are available at .

## Background

Since the beginning of the genomics era, numerous authors have warned against on-coming information overload, using metaphors that evoke natural disasters ("deluge," [1] "avalanche" [2], "tsunami" [3]) to emphasize how our capacity to generate data threatens to overwhelm our ability to deploy it in research. Finding ways to store and analyze vast amounts of data is not as difficult as it once was, thanks to improvements in database technologies, web services, and computer hardware, but developing graphical software that allows scientists to visualize, explore, and interact with novel and rapidly expanding data sets from genomics remains a challenging task.

Flexible, highly-interactive visualization software has great value in genomics because it enables scientists to explore the genomic landscape without knowing in advance what patterns and relationships they might find [4-6]. Visualization techniques provide an excellent complement to more abstract, quantitative or statistical analysis methods in that they rely on innate human visual processing systems, rather than on abstract mathematical reasoning. Discoveries arising from interactive inspection of the genomic landscape will always require corresponding statistical validation, but visual methods have tremendous power to expose compelling patterns and give scientists ideas on what to test.

Developing visualization software for genomics can be difficult. Genome science changes so rapidly that new data types frequently appear in advance of statistical methods and visualization software needed to analyze and display them. Conceiving effective ways to represent new types of biological data in graphical formats can be difficult in part because even the scientists generating the data may not know precisely what they want to see until they have seen and interacted with real-life examples. Once they do, the experience of viewing their data in graphical format often suggests new questions and new ideas, creating the need to develop new ways to display data and new modes of interaction. However, once a developer has created a working application for scientists to use, it may not be easy or even feasible to modify how the data are represented, depending on how the developer has implemented the graphical components.

The Genoviz Software Development Kit (SDK) aims to solve some of these problems. The Genoviz SDK is an open-source, freely available Java-based toolkit for building genomics visualization applications. It provides core functionality developers can easily deploy in their applications, notably interactive, dynamic zooming, which allows rapid navigation and exploration of genome-scale data sets covering many orders of magnitude, from chromosomes to genes to individual base pairs. The toolkit implements functionality well-suited to genomics visualization applications, but its architecture also makes it easy for developers to invent new ways to represent emerging data types in graphical formats. The Genoviz SDK aims to help developers build new applications iteratively and organically, inventing novel graphical representational schemes in collaboration with users as they explore their own data, make discoveries, and think of new questions for their software and experiments to address.

## Implementation

The Genoviz SDK is implemented using the Java programming language and requires Java versions 1.6 or above.

## Results

The Genoviz SDK is an object-oriented, Java-based graphics framework that provides methods, objects, and a class hierarchy for developers to display genomic data in two-dimensional fields. It originated as the Neomorphic Software Development Kit (NGSDK) and was first developed at Neomorphic Software, a bioinformatics company that later merged with Affymetrix, which continued development of the software. The NGSDK later entered the public domain as open source software under a new name: the Genoviz SDK. As a result, many classes and packages in the toolkit bear appellations "neo" and "affymetrix," reflecting the Genoviz SDK's origins at the two companies.

The Genoviz SDK's core graphics system employs three collaborating classes: Scene, a View, and Glyph. A Scene object represents a two-dimensional data field and its coordinate system. For example, a Scene might represent the physical map of a single chromosome or chromosome arm, with its associated annotations and sequence data. In this case, the coordinate system comprises nucleotide positions, most easily expressed as whole numbers. Another Scene might represent a genetic or cytological map; in this case, the coordinate system might be based on map units, which can have fractional values. To accommodate different types of maps, the trio of interacting objects use floating point numbers to indicate positions, but programmers are free to use either floating point numbers or integers when adding items to a Scene.

A View represents the user's current view on the Scene. It captures the user's current level of zoom and the range of visible coordinates. Each View references a single Scene and encapsulates algorithms for transforming coordinates referencing the Scene's biology-based coordinate system into the pixel-based coordinate system of the user's computer screen. The details of how Scene and View objects accomplish this translation are largely hidden from the programmer, and most programmers would not need to manipulate instances of these classes directly, unless they required specialized behavior not already provided as part of the toolkit. However, because the system is open source, programmers can investigate the transformation logic in detail when necessary.

**Figure 1:**
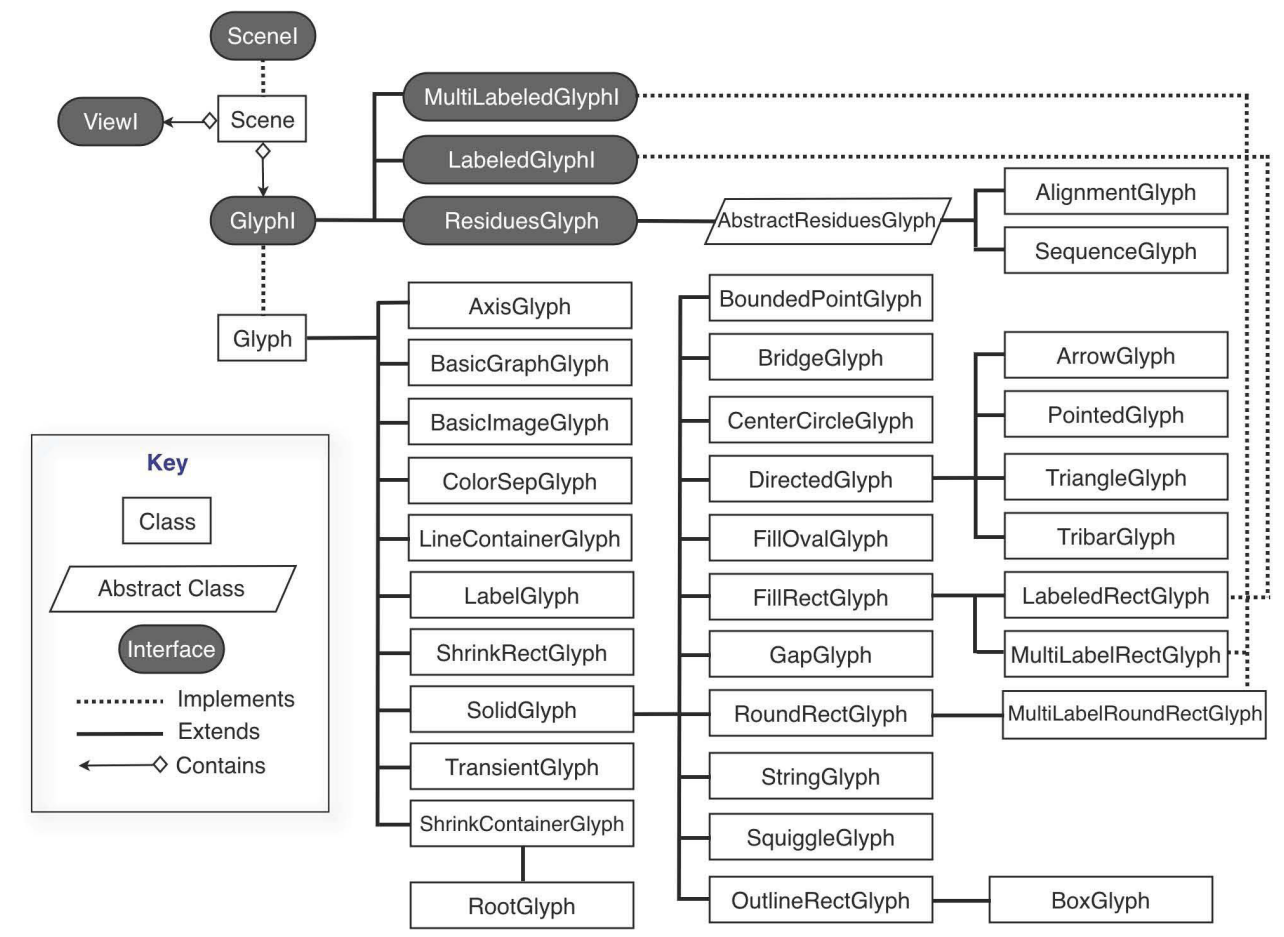
**Scene, View, and Glyphs**. The core graphics capability in the Genoviz SDK involves three core collaborating classes/interfaces. A Scene implements the SceneI interface and contains references to ViewI and GlyphI objects. In a typical application, there is usually only one ViewI per Scene, but Scenes can support multiple ViewI objects in order to present of alternative views on the same data. Scene objects also contain a reference to a single root GlyphI object, which in turn contains many children. When the View on a Scene changes, the Scene invokes the **draw** method of its associated GlyphI object, which then may recursively invokes the **draw** method of its child Glyphs.

**Figure 2:**
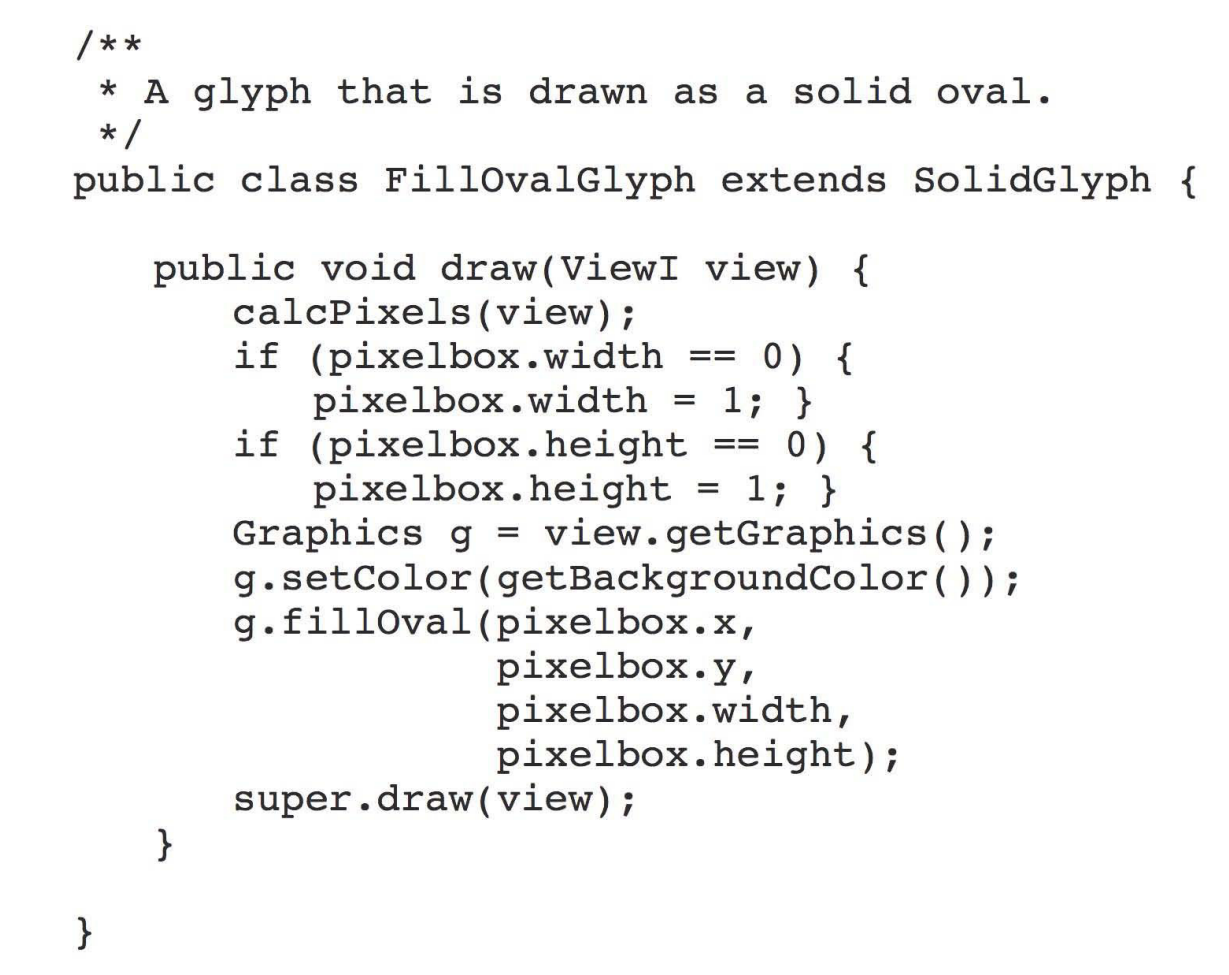
**A sample **draw** method for a simple Genoviz SDK Glyph class**. The method accepts a View object, defined as type ViewI, an interface containing View-related methods. A superclass' **calcPixels** method translates the Glyphs map-based coordinates (the coordbox) to pixel-based coordinates. The Java AWT Graphics object then draws an oval shape in the space bounding the Glyph's location in pixel coordinates.

Glyph objects are individual graphical elements that belong to a Scene and change their appearance depending on the scale of the View (or Views) in which they appear. A Scene may contain many thousands of Glyphs; typically, only a subset of these is on display at once. The toolkit contains a rich array of ready-to-use glyphs (see Figure 1,) and developers can create new Glyph classes by implementing the GlyphI interface or by subclassing existing GlyphI implementations. Glyphs are designed to know how to draw themselves; hence, a developer can exert near-total control over their appearance at different zoom levels. To create a new Glyph class with a novel appearance, the developer would over-ride the drawing routines promised in the GlyphI interface, perhaps using extant Glyph source code as a rough guide to how achieve different effects. (A simple example appears in Figure 2.) Glyphs can also be nested within other Glyphs in parent-child relationships, allowing for a variety of effects. For example, the drawing method of a parent (or container) Glyph might measure its available screen space and choose to invoke its child Glyphs' drawing methods only if there are enough pixels to display them adequately, an example of semantic zooming.

The Scene, Glyph, and View come together as components of a fourth object, called a Widget (see below), that manages their interactions with each other and implements basic functionality for mediating user interactions with the genomic data on display in the Scene. The Widget typically creates and manages scrollbars, sliders, or other graphical user interface components the user operates to zoom or pan the display. The Widget intercepts user requests to change scale or position, and then triggers invocation of drawing methods via the one or more View objects associated with its Scene. Each View in turn requests its Scene's Glyphs to draw themselves; Glyphs consult the View in which they appear to determine their sizes relative to the pixel-based coordinate system of the computer screen and then use the built-in Java AWT Graphics object (also obtained from a View) to draw graphical elements on the screen. The division of responsibility between Scene, Glyph, and View makes it possible for a single Scene to appear in multiple Views, allowing multiple, alternative representations of the same data. This can be useful in a number of settings, for example, when viewing an overview graphic of an entire chromosome in tandem with a zoomed-in view of the same data.

An important feature of this design is that it enables the graphics system to operate relatively independently of the data models, akin to the Model-View-Controller architectural design pattern commonly encountered in modern software applications that aim to separate business logic from presentation strategy [7]. Glyphs can contain references to custom data models, and specialized Glyph subclasses may implement drawing logic that consults these data models, but otherwise, Glyphs do not enforce a particular scheme for modeling genomic data.

One of the core features of Genoviz SDK is that the graphical rendering system handles zooming and panning (scrolling) without the programmer having to provide explicit control of the system in response to user drags on scrollbars or sliders attached to a display. The zooming sub-system typically uses a default (but replaceable) log-based Transform object to adapt the amount of zoom obtained per unit of user drag (e.g., the number of pixels a scrollbar thumb moves) to the current scale of the view on display in an application. As a result, drag gestures at high zoom change the scale of the display less than do drags at lower zoom. Similarly, the programmer does not typically have to determine the vertical positioning of Glyphs in horizontal maps. Typically, the widget component implements algorithms (encapsulated in Packer objects) that determine the vertical location of new Glyphs, stacking them in ways that prevent them from colliding in the two-dimensional data field.

### Custom data representation schemes using Glyphs

**Figure 3:**
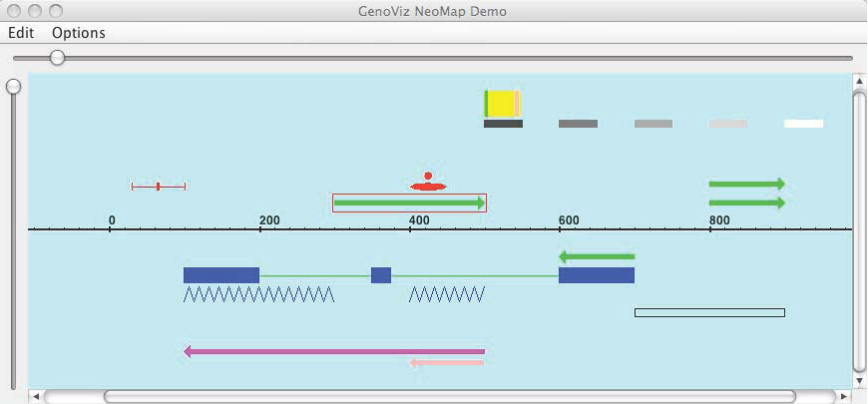
**Genoviz SDK Glyphs on display**. The toolkit contains Glyph classes programmers can use to develop new applications and new representation schemes for genomic data. The green arrow outlined in red is an instance of an ArrowGlyph. The outline indicates the user has clicked the Glyph and the Glyph is now considered selected. This example application is available from demo sub-directories within the genoviz sdk package in the Genoviz source code repository.

Programmers can easily implement novel visualization ideas within the Genoviz framework by implementing and deploying new Glyphs. Figure 3 presents a screen capture from a demonstration application containing several examples of Glyph types already implemented as part of the Genoviz SDK. Note that some are comprised of several other Glyphs, such as the central Glyph containing of three blue rectangular Glyphs. The basic Glyph class, which developers would typically sub-class, contains methods that allow a Glyph to recursively draw its children, using the coordinates of the parent Glyph to delimit the child Glyphs' available space. The ability to invoke this method recursively is particularly useful for representing compound features, such as the set of exons and introns that comprise a gene structure [6]. Because the toolkit is open source, developers can examine the source code and use these pre-existing Glyph classes as a rough guide to what types of effects are possible.

**Figure 4:**
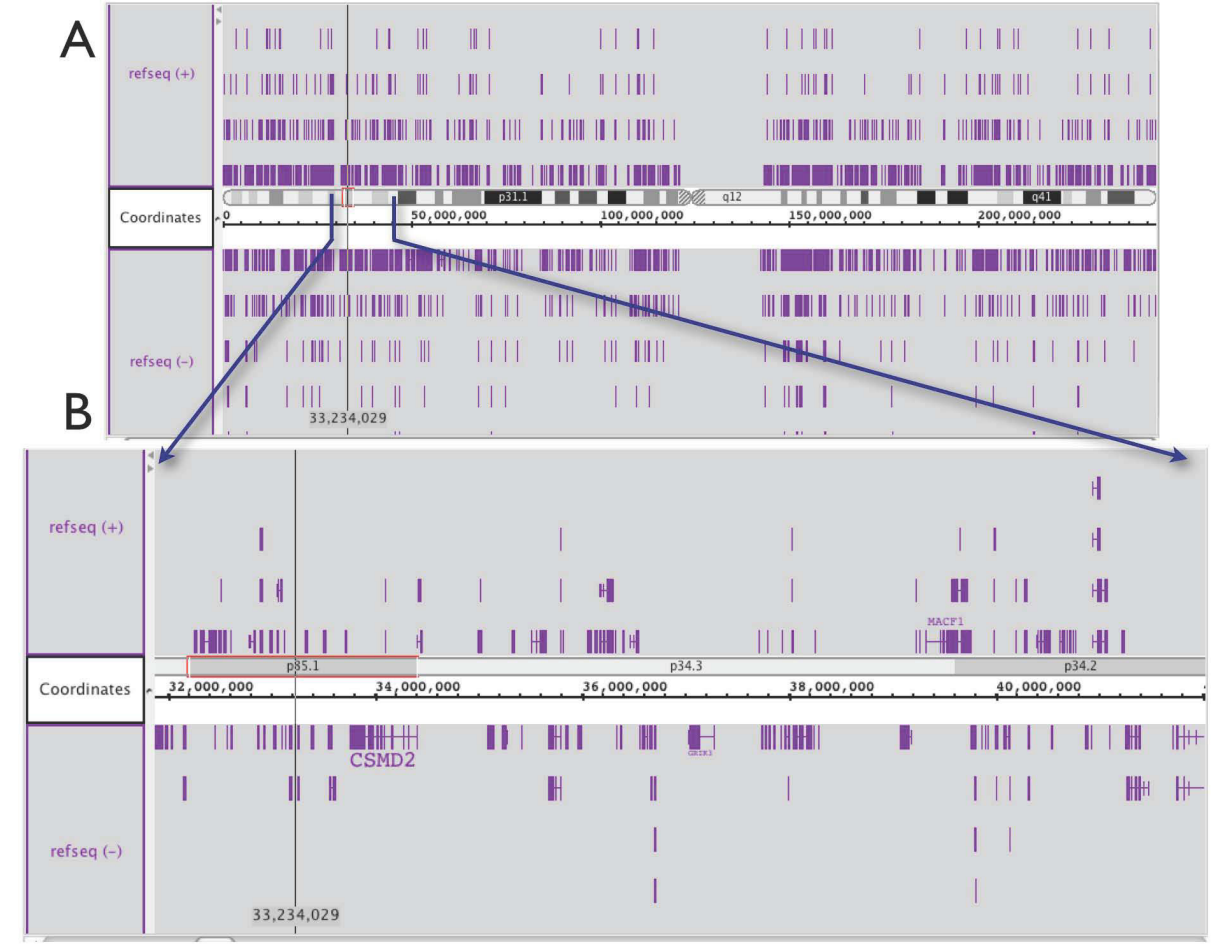
**Semantic Zooming and Child Glyphs**. The image below comes from the Integrated Genome Browser, which can display a chromosome ideogram alongside the genome axis. **A**. At low zoom, component bands merge with parent bands and labels appear only when the display size in pixels is large enough to accommodate them. **B**. At high zoom, chromosome bands resolve and labels appear. Glyph classes that implement this behavior are in the igb subdirectories within the Genoviz project source code repository at sourceforge.net.

**Figure 5:**
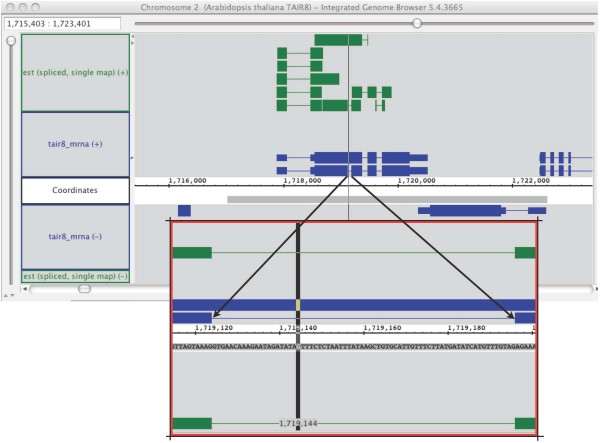
**Semantic zooming and sequence data**. This screen capture from the Integrated Genome Browser showing gene models from *Arabidopsis thaliana *and overlapping, spliced EST alignments shows two view on the same sequence Glyph. At low zoom (top,) the loaded sequence appears as a gray bar beneath the sequence axis. At high zoom, the gray bar resolves into characters (A, G, T, C) representing the DNA sequence bases.

Programmers can design new Glyph classes that implement semantic zooming, a form of zooming in which objects change their representation depending on the zoom level and which has special relevance in genomics where the scale of the data ranges across several orders of magnitude, from entire chromosomes to individual base pairs. Developers can modify zooming behavior by over-riding the Glyph "draw" method, as discussed above. A Glyph accesses its currently available screen space via a View, passed as a parameter to the Glyph's drawing routines, and then change its drawing logic accordingly. For example, a Glyph might choose to draw its label based on whether or not there is enough horizontal space to accommodate the label text. Similarly, a Glyph might choose to invoke the drawing routines on its child Glyphs only when the parent Glyph achieves a pre-determined size in pixel-based coordinate system of the user's display. Other, more complex behaviors are possible. Figures 4 and 5 present an examples of semantic zooming from the Integrated Genome Browser [8], a Genoviz SDK-based application. In Figure 4, the developer has created a Glyph representing the cytological map of the human genome. At low zoom, the entire chromosome occupies the View, while at higher zoom, individual labeled bands comprising the cytological map become visible as more space becomes available. Figure 5 presents another example of semantic zooming from the IGB software. The software implements partial (also called "lazy") loading of sequence data from a back end data server, an optimization scheme that lets the user access regions of interest without having to download an entire genome or chromosome sequence, which could be multiple megabytes or even gigabytes. At low zoom, regions where sequence has already loaded appear in gray, tagging regions the user has already examined in depth. At high zoom, these regions resolve into letters representing base pairs.

### Separation of Concerns: Graphics Semantics and Genomics Semantics

The Genoviz SDK aims wherever possible to separate the semantics of graphical rendering from the semantics of the genomic data. This design decision enables developers to reuse preexisting libraries and applications when creating visualization applications. The Genoviz SDK graphics logic does not specify how data should be represented internally within an application. At first glance, this statement may seem contradictory in that the Genoviz SDK aims to make creation of genomic data visualization applications easy and convenient for programmers. However, the precise semantics of genomic data models are often application-specific, whereas the graphics components more often generalize across diverse applications and problem domains. For example, a developer who has implemented a database system for representing genomic data may wish to use data models that harmonize with the database. Similarly, a developer familiar with the open source BioJava library might prefer to use BioJava data models in conjunction with the Genoviz SDK [9]. To ensure maximum reusability, the Genoviz SDK does not require programmers to conform to any pre-determined scheme for representing genomic data. By separating drawing logic from genomic semantics of genes and genomes, sequence and annotations, the Genoviz SDK graphics system provides conveniences for the programmer that are nonetheless well-adapted to representation of genomic data.

### Genoviz SDK Widgets

The Genoviz SDK includes several classes (called Widgets) that provide convenient functions for interacting with and representing data types commonly encountered in bioinformatics. These Widgets implement a generic interface that specifies basic functionality related to zooming, panning, selection, and interaction with the underlying graphics system. Widgets provide methods for establishing horizontal and/or vertical axes, setting display bounds, panning, zooming, selecting and placing items at designated positions, configuring glyph factories and data adapters (for creating graphical objects with common attributes), setting background colors, establishing window resize behavior, and managing event handler objects that intercept and respond to user interactions. Specialized Widget implementation classes also provide methods and functionality for representing specific categories of genomic data. Some of the currently available widgets include a NeoMap object, for representation of physical and genetic maps; a TieredNeoMap widget for display of physical or genetics map data in individually-adjustable and configurable tracks; a NeoAssembler widget, aimed at display of EST/cDNA assemblies; and a NeoSeq widget for display of sequence data. By providing a generic Widget interface, the Genoviz SDK framework aims to encourage developers to create new widgets that support emerging data types, such as data from sequence-based expression profiling.

**Figure 6:**
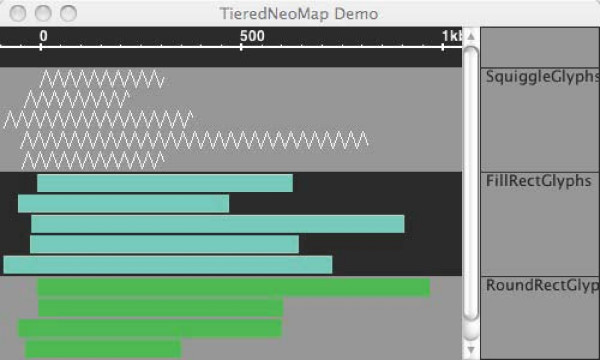
**TieredNeoMap**. The TieredNeoMap is a specialized subclass of NeoMap that allows developers to display data in labeled tracks. Typically, this functionality is used to segregate data sets arising from different sources so that users can compare them easily.

The NeoMap and TieredNeoMap widgets are used to place annotations, sequence, and functional genomics data sets such as chIP-chip data into a linear coordinate system. Their primary use is to support implementation of genome browser-like applications, where items of interest appear alongside a genomic sequence axis representing base pair positions. As such, they include functions that allow selection of items based on their position inside the map coordinate system and contain layout algorithms (implemented in Packer objects) that determine where items are located in the vertical dimension. A developer can add new Glyphs to a NeoMap or TieredNeoMap by invoking addItem methods that accept start and end coordinates and specialized factory objects that instantiate new Glyphs using pre-determined styles. NeoMap and TieredNeoMap widgets handle integer- and real-number-based coordinate systems, thus making them useful for display of cytological or genetic maps, as well as physical, base-pair maps. The TieredNeoMap (Figure 6) is a NeoMap that can position Glyphs into separate tiers (tracks) the user can move, hide, collapse, or transform.

**Figure 7:**
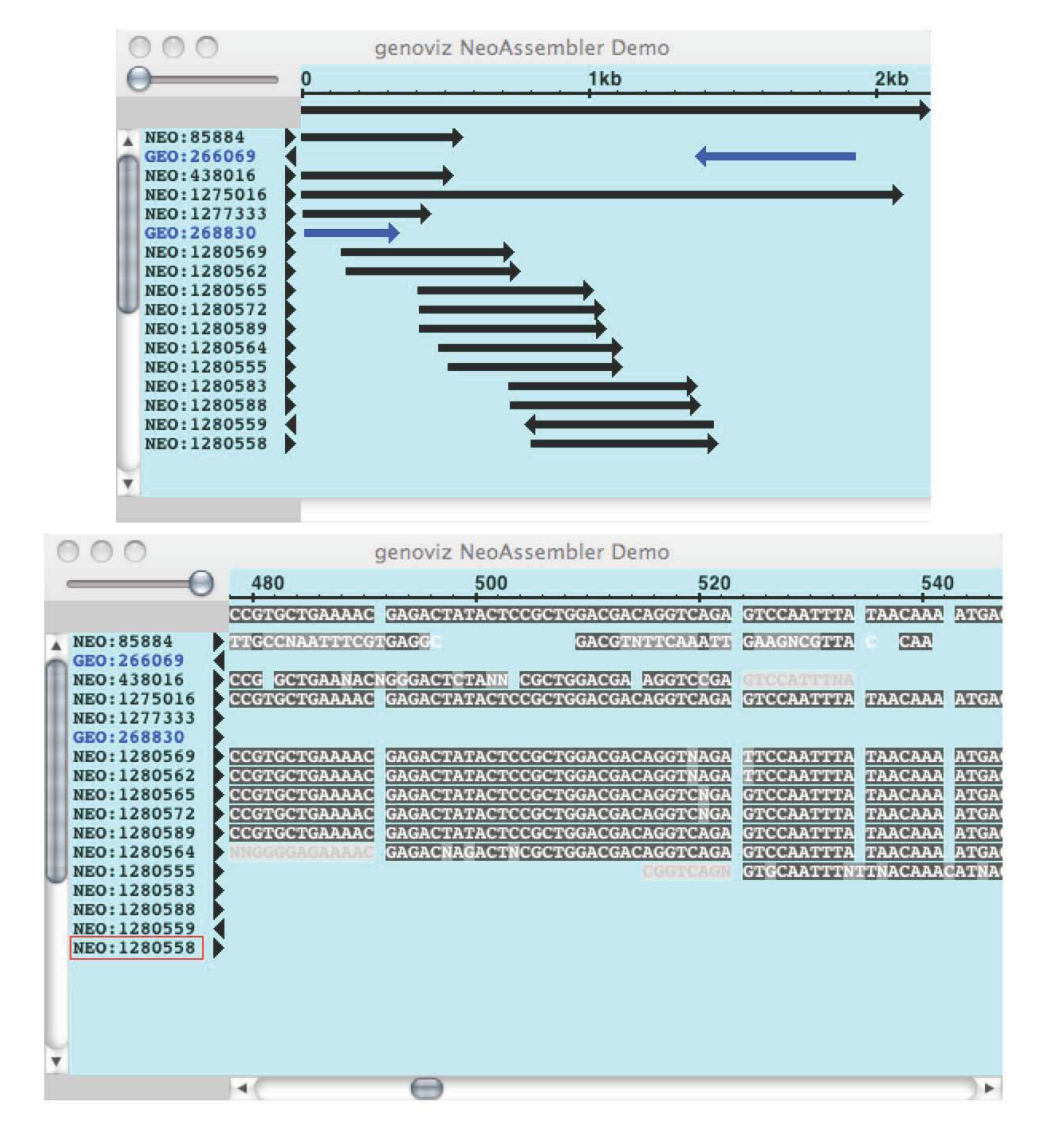
**NeoAssembler**. The NeoAssembler widget displays assembled sequences and their merged consensus sequence.

**Figure 8:**
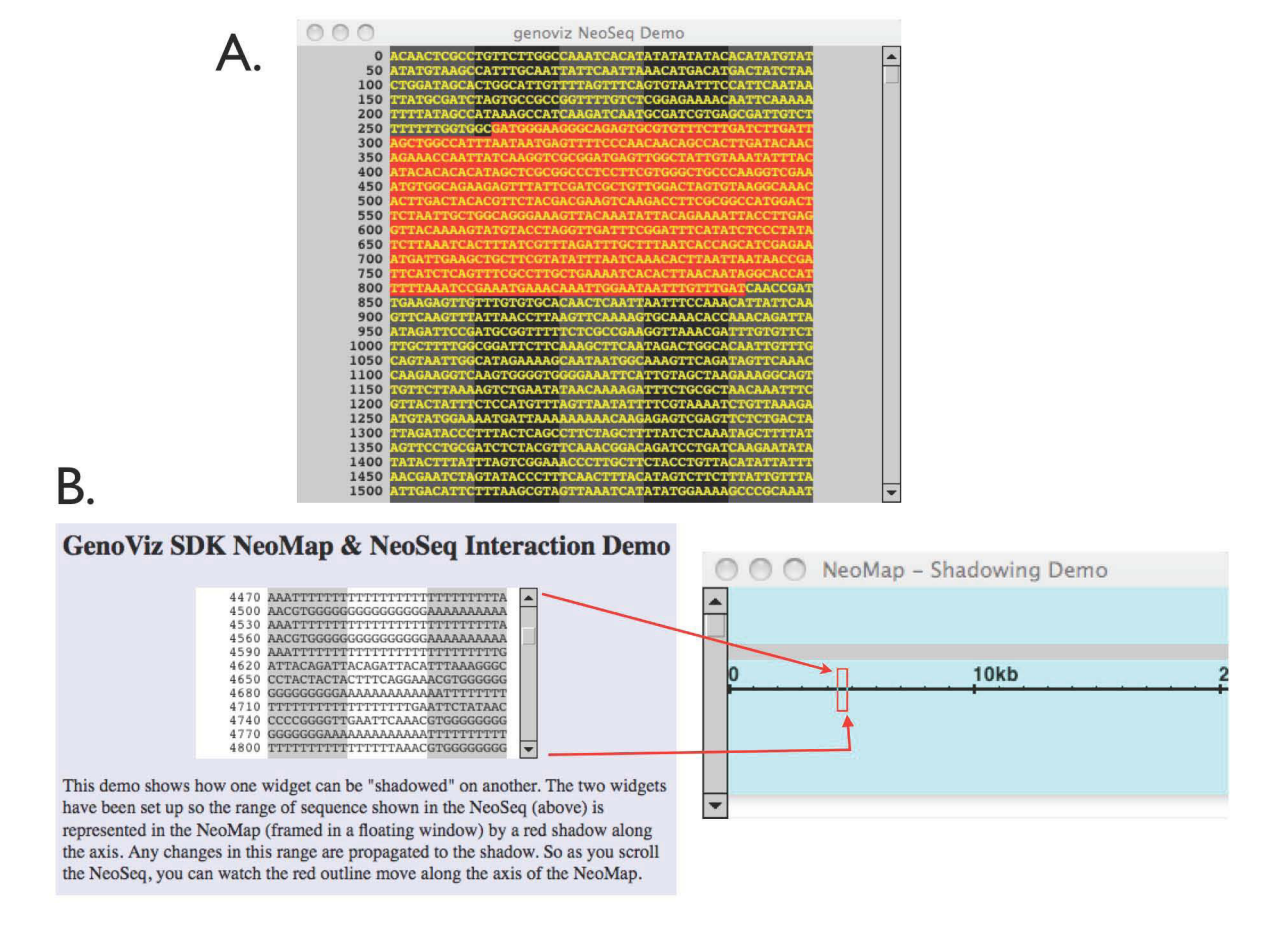
**NeoSeq and NeoMap widgets can work in concert**. (**A**) A NeoSeq widget displays sequence as characters. Here, the user has highlighted a section of the sequence. The selected region appears in red. (**B**) NeoSeq and NeoMap working in concert. Here, a red RectangleGlyph covers the section of the NeoMap corresponding to the section of sequence displayed in the NeoSeq. Click-dragging the RectangleGlyph in the NeoMap scrolls the sequence displayed in the NeoSeq. Likewise, scrolling the NeoSeq moves the RectangleGlyph. In many settings, it is useful to be able to view sequence data in a scrollable window, linked to a map in which annotations and other notations on the sequence appear.

As with the NeoMap and TieredNeoMap, the NeoAssembler widget (Figure 7) includes convenience methods for a specialized data type: sequences assemblies. The NeoAssembler displays short-read sequences (e.g., ESTs or genomic sequence reads) in rows beneath a typically much-longer consensus sequence and includes methods that allow the programmer to specify how mismatches and other aspects of the assembly will appear to the user. As with the other widget objects, the NeoAssembler provides ways for the user to navigate the data scene on display. At high zoom, sequences resolve into letters representing base pairs; at low zoom, they appear as annotated, rectangular Glyph objects that show the large-scale structure of the assembly, including mismatch positions and directionality of reads relative to the consensus. The NeoSeq widget (Figure 8) contains functions aimed at convenient display of a single sequence. It supports text-based display of sequence data in a scrollable display and supports user interactions relevant to the representation of sequence as simple text, such as selection of subsequence, highlighting, and copy-and-paste.

### Creating an application using the Genoviz SDK

A typical Genoviz SDK application consists of several collaborating classes: parsers that read data from files or databases and generate in-memory data structures; adapter objects that translate these data structures into Glyph objects shown in the display; one or more display components (e.g., NeoMap) that mediate user interactions with data, and custom application logic that specifies how the application will respond to user interactions with Glyphs, menus, scrollbars, and other graphical elements. Typically, developers attach data models representing genomic data to Glyph objects via a generic setInfo method, which accepts any Java object; this allows developers to link the object models representing genomic data to the graphical elements that control how the data will appear within a display. When users interact with Glyphs, the display component generates events, which the application logic may capture and then interpret. For example, right-clicking a Glyph representing a gene model might signal a request to get more information about it, as happens in the Integrated Genome Browser [8]. Alternatively, it could represent a request to perform an editing operation on the underlying genomic data model the Glyph represents. In this way, the graphics system provides a visual representation of data structures that users can easily inspect and manipulate via Glyph objects, similar to windows, checkboxes, and other familiar graphical elements that users can click, drag, and manipulate in standard graphical user interfaces.

## Discussion

Developers evaluating the Genoviz SDK for use in their applications may be interested in comparing it to other graphics frameworks that render two-dimensional data. The Jazz/Piccolo framework, first developed at the University of Maryland, enjoys a large group of enthusiastic users [10]. In Jazz/Piccolo, zooming is typically two-dimensional, imitating the action of camera rising and lowering over a two-dimensional data field. Jazz/Piccolo zooming involves focusing on a single point around which the data field contracts or expands as the user zooms in or out. In genomic applications, where the field of data is typically one-dimensional and involves stretching and contracting a genomic sequence axis, zooming is simpler and more intuitive when restricted to a single dimension [6]. The graphical components implemented in Jazz/Piccolo require more memory and processing power than their equivalents in the Genoviz SDK, thus limiting their usefulness for display of vast genomic data sets [11]. They are best-suited to the representation of hundreds of data objects, whereas the Genoviz SDK is well-suited for representation of genome-scale data sets, which can include hundreds of thousands of objects and millions of data points.

Other toolkits for genomics visualization that have been published include bioTK and bioWidgets. David Searls' bioTk toolkit offered a set of configurable graphical components, termed "widgets," that programmers could manipulate using the Tcl/Tk command-line language [12]. This early toolkit included components for drawing chromosomes, genome maps, and sequence displays. Later, Gregg Helt developed bioTK-Perl, which allowed Perl developers to use similar components in Perl applications, such as Genotator, a workbench for genome annotation developed by Nomi Harris [13]. At least two Java-based toolkits for genomics visualization were developed in the late 1990s, including the BioViews toolkit from the Berkeley Drosophila Genome Project [5] and the BioWidgets toolkit from CBIL at the University of Pennsylvania [14]. To our knowledge, these toolkits are no longer supported. The Genoviz SDK draws inspiration from these early groups' work; however, its structured graphics approach more closely resembles the Jazz/Piccolo toolkit and places greater emphasis on efficient memory usage. The open source BioJava and BioPerl projects include sequence visualization components, but they are more tightly coupled to their respective BioJava and BioPerl data models [9].

**Figure 9:**
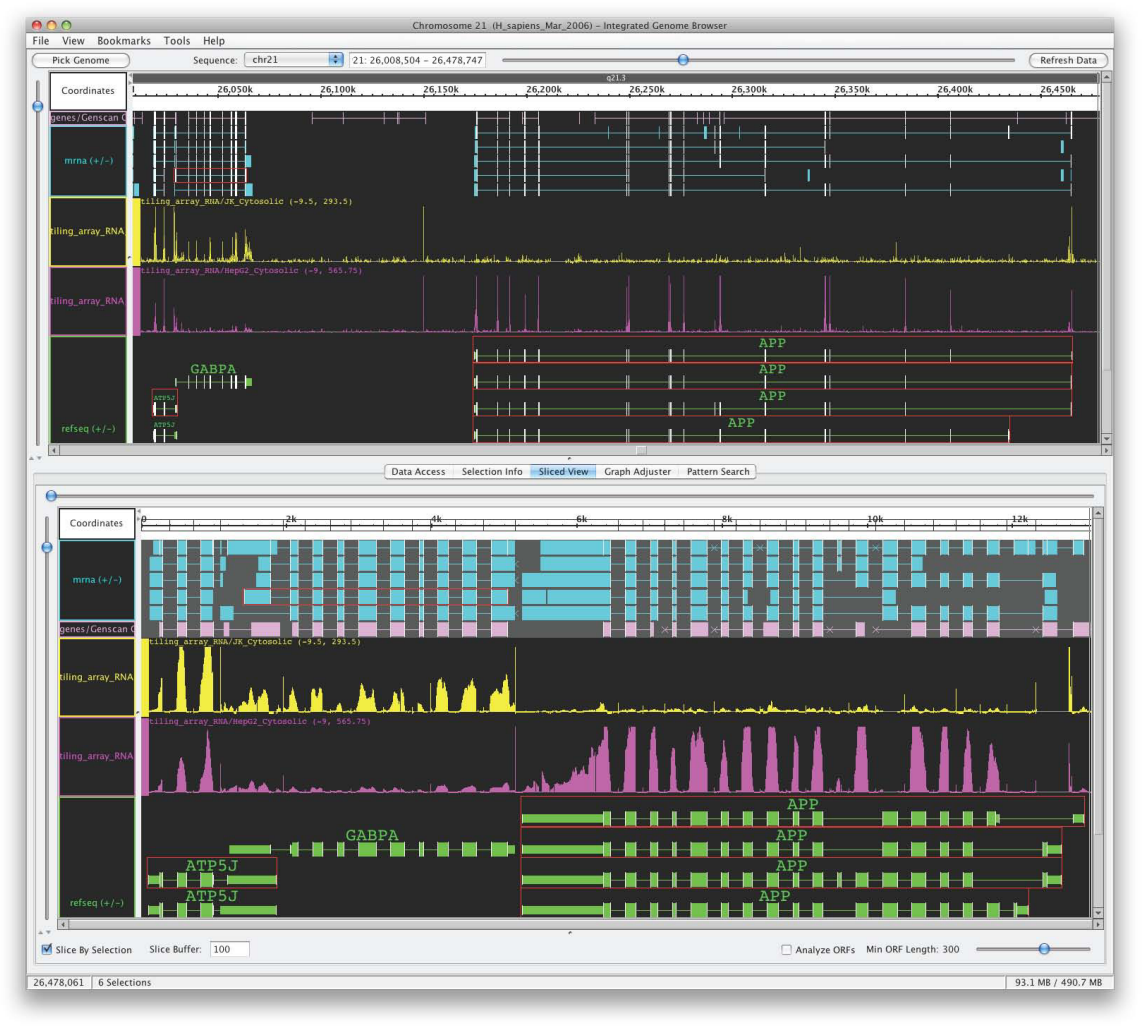
**Semantic zooming and other functionality implemented in the Integrated Genome Browser, version 5.3**. The top view shows a region of human chromosome 21 with gene annotations and mRNA alignments. The display also shows plots of expression array data from Jurkat and HepG2 cell lines, represented as a score for each tiling array probe chromosomal position. The bottom view shows a "sliced" subset of the same data, in which exons for selected annotations (outlined in red) along with flanking sequence have been spliced to form a virtual sequence and all annotations remapped to the virtual sequence. There are roughly 50,000 data points per expression array plot in the region displayed in the top view, averaging 50 data points per pixel along the horizontal axis. The graph Glyph object used to render these plots appears in both views. The display uses semantic zooming to render them differently according to the different resolutions. In the lower resolution top view, for each horizontal pixel position a vertical line is drawn representing the score range for data points whose genomic coordinates fall within that pixel. A slightly brighter dot is also drawn for the scores' average. In the sliced view at the lower part of the display, the resolution is high enough that each scored position is rendered as a simple bar. These rendering techniques can allow real-time visualization of more data than is shown here. For example, the two plots shown each have greater than 3 million data points on chromosome 21.

Several groups in industry and academia have implemented applications using the Genoviz SDK. These include Affymetrix, where co-authors Gregg Helt with Ed Erwin developed early versions of the Integrated Genome Browser (Figure 9), also available in the same sourceforge project as the toolkit itself. In late 2008, another group released the Genome Environment Browser, which used the Genoviz SDK to implement an interactive genome environment for users to explore genome-scale data sets, primarily tiling array data [15]. Other applications developed using the Genoviz SDK (and its earlier incarnation the Neomorphic Genome Software Development Kit) include the Neomorphic Annotation Station [6], which The Institute for Genomic Research commissioned to support their curation of the *Arabidopsis thaliana *genome [16], as well as proprietary genome display tools used and developed at Celera Genomics.

## Conclusion

The Genoviz SDK is a Java data visualization toolkit for genome data application developers. It handles the low-level aspects of linking object-oriented data models to graphical widgets so that application developers can focus on the unique aspects of their data and application logic, rather than implement graphical rendering algorithms. An award from the National Science Foundation (U.S.A.), as well as volunteer efforts from a small but growing community of developers, continue to support development of the IGB software and the Genoviz SDK. Major efforts currently underway include creation and updates of documentation for novice and experienced developers, creation of new tutorials showing programmers how they can use Genoviz SDK to add visualization capability to their applications, and development of demonstration applications showing Genoviz SDK graphics capability. Resources for developers and users alike are available from .

## Availability and Requirements

The Genoviz SDK is implemented in the Java programming language and is freely available under the Common Public License, v1.0, an Open Source Initiative  approved open source license. The project home page is , from where users can download and view source code anonymously. Users interested in downloading a pre-compiled copy of the Integrated Genome Browser software can obtain it at . Please note that the igb.bioviz.org site systems administrator compiles composite usage statistics aimed at tracking the overall number of downloads and average number of accesses per IP address. The sourceforge site also tracks general usage statistics. Other than this, no details about individual users and their visits to the site are tracked. To run the software, users will require Java version 1.6 or higher.

## Authors' contributions

All authors contributed development of Genoviz SDK source code, documentation, or both. All authors participated in writing or editing the manuscript. All authors read and approved the final manuscript.
